# bmVAE: a variational autoencoder method for clustering single-cell mutation data

**DOI:** 10.1093/bioinformatics/btac790

**Published:** 2022-12-07

**Authors:** Jiaqian Yan, Ming Ma, Zhenhua Yu

**Affiliations:** School of Information Engineering, Ningxia University, Yinchuan 750021, China; School of Information Engineering, Ningxia University, Yinchuan 750021, China; School of Information Engineering, Ningxia University, Yinchuan 750021, China; Collaborative Innovation Center for Ningxia Big Data and Artificial Intelligence Co-founded by Ningxia Municipality and Ministry of Education, Ningxia University, Yinchuan 750021, China

## Abstract

**Motivation:**

Genetic intra-tumor heterogeneity (ITH) characterizes the differences in genomic variations between tumor clones, and accurately unmasking ITH is important for personalized cancer therapy. Single-cell DNA sequencing now emerges as a powerful means for deciphering underlying ITH based on point mutations of single cells. However, detecting tumor clones from single-cell mutation data remains challenging due to the error-prone and discrete nature of the data.

**Results:**

We introduce bmVAE, a bioinformatics tool for learning low-dimensional latent representation of single cell based on a variational autoencoder and then clustering cells into subpopulations in the latent space. bmVAE takes single-cell binary mutation data as inputs, and outputs inferred cell subpopulations as well as their genotypes. To achieve this, the bmVAE framework is designed to consist of three modules including dimensionality reduction, cell clustering and genotype estimation. We assess the method on various synthetic datasets where different factors including false negative rate, data size and data heterogeneity are considered in simulation, and further demonstrate its effectiveness on two real datasets. The results suggest bmVAE is highly effective in reasoning ITH, and performs competitive to existing methods.

**Availability and implementation:**

bmVAE is freely available at https://github.com/zhyu-lab/bmvae.

**Supplementary information:**

Supplementary data are available at *Bioinformatics* online.

## 1 Introduction

Cancer develops by accumulating genomic mutations (Nowell, 1976), and this evolutionary process can be depicted by a phylogenetic tree where distinct tumor clones appear at different nodes of the tree (Ross and Markowetz, 2016). Each clone is characterized by a set of genomic mutations, and genetic differences among tumor clones are known as intra-tumor heterogeneity (ITH) (Stratton *et al.*, 2009; Swanton, 2012). ITH acts as one of the important factors leading to drug resistance and cancer relapse, therefore accurately deciphering ITH from genome data is essential for personalized cancer therapy. Conventional bulk sequencing has been widely used for ITH profiling (Eaton *et al.*, 2018; Satas and Raphael, 2017), while it requires a deconvolution step to identify all clones from the averaged signal of thousands or even millions of cells, which often results in low-resolution indication of the ITH due to deficient coverage of low-prevalence clones. By comparison, single-cell DNA sequencing (scDNA-seq) (Gawad *et al.*, 2016) is a powerful technique to profile genomic variations at single-cell resolution, and particularly useful to decipher the ITH and evolutionary history of tumor based on single-cell mutation data (Kuipers *et al.*, 2017).

Despite to its advantage of providing high resolution indication of the ITH, scDNA-seq has some technological factors that complicate the downstream data analysis. Whole-genome amplification involved in scDNA-seq often results in unbalanced amplification of alleles and even allele-dropout (ADO) (Navin, 2014). ADO has a significant effect on heterozygous mutation sites, where one or both of the alleles are not adequately amplified, and this often yields false negative (FN) calls for heterozygous mutations (Gawad *et al.*, 2014; Hou *et al.*, 2012). In addition, sequencing errors impose a challenge of distinguishing true mutations from technological background noise, and often result in false positive (FP) calls (Hou *et al.*, 2012; Xu *et al.*, 2012). ADO and uneven coverage also cause unobserved sites (Hou *et al.*, 2012). Another issue related to scDNA-seq data is cell doublet that represents mixed profiles of two or more cells (Zafar *et al.*, 2017). These critical issues give rise to a challenge of accurately decipher the ITH and tumor evolutionary history.

Many computational methods have been developed in recent years to infer tumor phylogenetic tree from single-nucleotide variations of cells. These methods are typically classified into three categories based on the adopted evolutionary model: infinite-sites model (ISM), Dollo parsimony model and finite-site model (FSM). ISM takes a strict evolutionary assumption that each mutation can be gained at most once and the acquired mutations will not be lost during the evolutionary process. For instance, SCITE (Jahn *et al.*, 2016) employs a Markov chain Monte Carlo-based approach to reconstruct tumor mutation tree under the ISM assumption, and PhISCS-BnB (Sadeqi Azer *et al.*, 2020) uses a branch and bound algorithm to deliver perfect phylogeny. To relieve the constraint of the ISM, Dollo parsimony model allows each mutation to be lost multiple times. Popular methods built on the Dollo model include SPhyR (El-Kebir, 2018), SASC (Ciccolella *et al.*, 2021b) and GRMT (Yu *et al.*, 2021). To further relax the constraint on the evolutionary model, FSM allows both parallel evolution and back mutation. For instance, SiFit (Zafar *et al.*, 2017) uses a Markov chain Monte Carlo-based method to reason cell lineage tree following FSM, and CellPhy (Kozlov *et al.*, 2022) employs a finite-site Markov genotype model to infer tumor phylogenetic tree. These methods perform well on small datasets, while their scalability may be limited on large datasets containing thousands of cells due to huge search space of the tumor evolutionary tree. One feasible solution to simplify phylogeny inference is to cluster cells or mutations before tree reconstruction, where clustering of cells or mutations is adopted as a preprocessing task to shrink the search space of tree structures, thus improves the efficiency of phylogeny inference.

To date, several bioinformatics methods have been proposed to cluster single-cell mutation data (Borgsmüller *et al.*, 2020; Chen *et al.*, 2020; Myers *et al.*, 2020; Ross and Markowetz, 2016; Roth *et al.*, 2016; Yu *et al.*, 2022; Yu and Du, 2022; Zafar *et al.*, 2019). These methods can be divided into two classes: detecting tumor clones with or without phylogeny inference. Clonal tree depicts the lineage relationship between distinct tumor clones, and can be treated as a ‘regularizer’ on the mutational states of clones. OncoNEM (Ross and Markowetz, 2016) employs a heuristic algorithm and SiCloneFit (Zafar *et al.*, 2019) uses a Bayesian framework to jointly infer clonal structure and phylogeny. RobustClone (Chen *et al.*, 2020) first recovers genotypes of single cells based on robust principal component analysis, and then reconstructs clonal tree using the Louvain–Jaccard clustering method (Levine *et al.*, 2015). A recent method called AMC (Yu and Du, 2022) maps mutations into distinct clusters, and reasons tumor phylogeny based on the mutation clusters. celluloid (Ciccolella *et al.*, 2021a) aims to address the similar problem of mutation clustering, but does not provide clonal genotypes. These methods show good performance on small datasets, but may suffer from low efficiency when dealing with complex clonal architectures. Clustering cells without phylogeny inference forms another paradigm for detecting tumor clones. For instance, SCG (Roth *et al.*, 2016) leverages variational inference to estimate clonal composition and genotypes. BnpC (Borgsmüller *et al.*, 2020) employs a Bayesian model to jointly infer tumor clones and their genotypes, while it suffers from high computational complexity especially on large datasets (Yu and Du, 2022). SBMClone (Myers *et al.*, 2020) uses a biclustering method to identify blocks of cells and blocks of mutations from sparse mutation data and can deal with dataset containing tens of thousands of single-nucleotide variations. One disadvantage of SBMClone is that it does not output genotypes of inferred clones. SCClone (Yu *et al.*, 2022) employs an expectation–maximization algorithm to jointly infer tumor clones and their mutational states, but suffers from high computational complexity on large datasets. Taken together, clustering high-dimensional single-cell mutation data is a hard challenge, and more efficient and accurate methods are still needed for scDNA-seq mutation data.

In this article, we introduce a novel method called bmVAE to cluster single-cell mutation data based on a variational autoencoder (VAE). VAE combines unsupervised deep learning with Bayesian inference and acts as an important type of generative models (Bi *et al.*, 2022; Goodfellow *et al.*, 2020; Higgins *et al.*, 2017; Kingma and Welling, 2013). It has been widely used to project single-cell RNA sequencing (scRNA-seq) data into a latent space where single cells are clustered (Grønbech *et al.*, 2020; Mitra and MacLean, 2021; Rashid *et al.*, 2021; Svensson *et al.*, 2020). For instance, Dhaka (Rashid *et al.*, 2021) uses a VAE model to unmask tumor heterogeneity from copy number alterations or gene expression data of single cells. The intuition behind our modeling of single-cell mutation data using a VAE-based approach is that the observed mutational signatures actually result from some underlying biological processes related to tumor evolution, and these processes are represented as distribution over the latent space (Rashid *et al.*, 2021). bmVAE first employs a VAE model to learn latent representation of each cell in a low-dimensional space, then uses a Gaussian mixture model (GMM) to find clusters of cells, finally uses a Gibbs sampling-based approach to estimate genotypes of each subpopulation. To the best of our knowledge, bmVAE is the first method that employs VAE model to scDNA-seq data for dimensionality reduction. We comprehensively compare bmVAE to the state-of-the-art methods on both simulated and real datasets, and the results show our method is an effective complementation to current arsenal of methods for detecting tumor clones from scDNA-seq mutation data.

## 2 Materials and methods

**Fig. 1.**
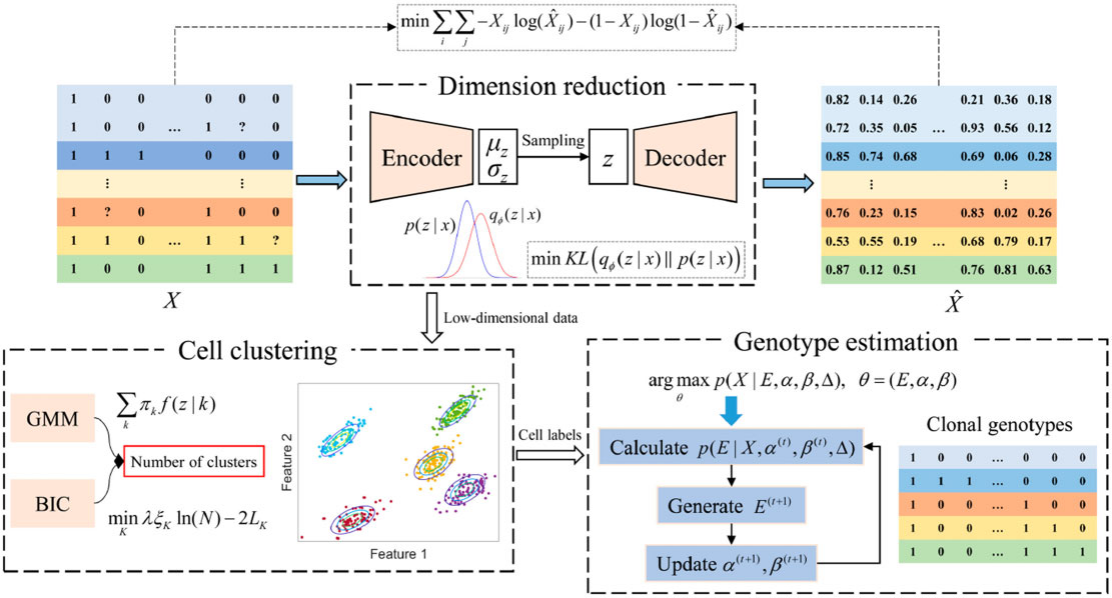
The framework of bmVAE. The workflow of bmVAE consists of three steps: dimensionality reduction, cell clustering and genotype estimation. bmVAE first uses a VAE to infer the low-dimensional representation of each cell by minimizing the Kullback–Leibler divergence loss and reconstruction loss (measured using cross-entropy). Based on the latent features of cells, GMM is employed to cluster cells into distinct subpopulations each of which has a different complement of mutations. Given the inferred cell-to-cluster assignments, Gibbs sampling method is used to estimate the genotypes of each subpopulation based on the input binary mutation data

The framework of bmVAE consists of three modules: dimensionality reduction, cell clustering and genotype estimation (as shown in Fig. 1). For dimensionality reduction, we use the VAE to project mutation data of each cell into a low-dimensional space. Based on the learned latent representation, cells are clustered into distinct subpopulations via the GMM and the optimal number of clusters is selected using Bayesian information criterion (BIC). Given the labels of cells inferred by the GMM, the Gibbs sampling method is used to reason the genotypes of each subpopulation. The following sections provide detailed descriptions about the three modules of bmVAE.

### 2.1 VAE for dimensionality reduction

Given a binary genotype matrix *X* of *N* cells at *M* genomic loci, we aim to learn an effective low-dimensional representation *Z* of *X* through unsupervised learning via the VAE model. The VAE consists of an encoder ϕ to deduce the conditional distribution $q_{\phi }(z|x)$ of latent representation *z* given input *x* (here *x* denotes the mutational profile of a cell and is recorded as one row of *X*), and a decoder *θ* to reconstruct *x* from *z*. Instead of representing the input data under an unconstrained manner, we impose a regularization constraint on the distribution of latent variables. Specifically, we assume *z* follows a multivariate Gaussian that has a diagonal covariance matrix, i.e. $z|x∼N(z;\mu_{z},\sigma_{z}^{2}I)$. This assumption is reasonable as distinct tumor clones have different mutational patterns that are characterized by the underlying tumor phylogenetic tree. The mutational patterns result from some biological processes related to tumor evolution, and these processes are modeled as the latent variables that are assumed to be independent with each other. A forward propagation of the VAE can now be described as: (i) the encoder ϕ takes *x* (the missing elements of *x* are simply set to 0) as input and outputs the means *μ_z_* and variances $\sigma_{2}^{2}$ of the normal distributions; (ii) the latent representation *z* is sampled from the distribution $N(z;\mu_{z},\sigma_{z}^{2}I)$; and (iii) the decoder *θ* takes *z* as input to reconstruct the input *x*.

The VAE model is trained to minimize the reconstruction loss $L(x,\hat{x};\phi ,\theta )$ under the regularization constraint applied on the posterior distribution $q_{\phi }(z|x)$, where $\hat{x}$ is the output of the decoder. Formally, the optimization problem is formulated as follows:

(1) $$\underset{\phi ,\theta }{\min}\lambda \cdot D_{KL}\left(q_{\phi }(z|x)||p_{\theta }(z)\right)+L(x,\hat{x};\phi ,\theta ),$$

where *λ* is a weight factor, $D_{KL}(\cdot ||\cdot )$ denotes the Kullback–Leibler divergence and $p_{\theta }(z)$ represents the true posterior of *z*. To make sure gradient descent algorithm can be used to train our model, we employ the reparameterization trick proposed in Kingma and Welling (2013) to make the optimization function differentiable (we do not directly sample *z* from the posterior but generate *z* with $z=\mu_{z}+\sigma_{z}⊙\epsilon$, where *ϵ* is sampled from the standard normal distribution). This reformulation enables the gradient can be backward propagated for updating the model parameters.

For binary mutation data, we treat each element of $\hat{x}$ as the probability parameter of Bernoulli distribution, and regard corresponding element of *x* as the observation, then the loss function $L(x,\hat{x};\phi ,\theta )$ can be defined as:

(2) $$L(x,\hat{x};\phi ,\theta )=-\sum_{j=1}^{M}x_{j}\;\text{log}({\hat{x}}_{j})+(1-x_{j})\;\text{log}(1-{\hat{x}}_{j}),$$

where *M* is the number of mutations, and we apply cross-entropy loss for each element of *x* (missing entries of *x* are not considered when calculating the loss). By calculating the mean loss for a batch of samples, we employ gradient descent algorithm to jointly update the parameters of encoder and decoder. After the model converges, each cell is passed through the encoder to get its latent representation $z=\mu_{z}$.

### 2.2 GMM for cell clustering

We use the GMM to cluster cells into distinct subpopulations based on the latent representations, and employ BIC to determine the best number of clusters. Formally, we begin with data homogeneity assumption (all cells come from same population), and iteratively increase the number of clusters until the minimum BIC value has not changed at least *κ* times (*κ* is set to 10 in this study) or a predefined maximum number of clusters is reached. This clustering procedure finally gives the labels of cells that can be used to infer the binary mutational profile of each subpopulation.

### 2.3 Gibbs sampling for genotype estimation

Cells originating from the same cluster share highly similar mutational profile, and inferring the underlying mutational profile of each cluster is an optimization problem over discrete space where the latent state is binary. As scDNA-seq mutation data are frequently confounded by FN and FP errors, parametric modeling of these errors is essential for accurately predicting the mutational states of tumor clones. Formally, we employ the Gibbs sampling to reason the posterior of the genotypes, and infer the maximum-likelihood estimations of the error rates. Suppose the inferred labels of cells are denoted by a vector $\Delta =(\delta_{1},\delta_{2},\ldots ,\delta_{N})$, *E* is a *K *×* M* binary matrix to represent the mutational states of *K* clusters, FP rate (FPR) and FN rate (FNR) are *α* and *β*, respectively, we calculate the posterior of *E_kj_* as follows:

(3) $$\begin{array}{c} p(E_{kj}=1|X,\alpha^{\left(t\right)},\beta^{\left(t\right)},\Delta )\\ =\frac{\prod_{i,\delta_{i}=k}p(X_{ij}|E_{kj}=1,\alpha^{\left(t\right)},\beta^{\left(t\right)})}{\sum_{c=0}^{1}\prod_{i,\delta_{i}=k}p(X_{ij}|E_{kj}=c,\alpha^{\left(t\right)},\beta^{\left(t\right)})} \end{array},$$

where we use *t* to denote the iteration index of Gibbs sampling, assume uniform prior for *E_kj_*, set both $\alpha^{\left(0\right)}$ and $\beta^{\left(0\right)}$ to 0.01. The conditional probability $p(X_{ij}|E_{kj})$ is defined as adopted in Roth *et al.* (2016):

(4) $$p(X_{ij}|E_{kj})=\left(\begin{array}{cc} p(0|0) & p(0|1)\\ p(1|0) & p(1|1) \end{array}\right)=\left(\begin{array}{cc} 1-\alpha & \beta \\ \alpha & 1-\beta \end{array}\right).$$

We then sample each *E_kj_* from the posterior to generate $E^{\left(t+1\right)}$. Given the mutational states of all clusters, the parameters *α* and *β* can be updated by maximizing the log-likelihood:

(5) $$\alpha^{\left(t+1\right)}=\frac{\sum_{i}\sum_{j}\left(1-E_{\delta_{i}j}^{\left(t+1\right)}\right)X_{ij}}{\sum_{i}\sum_{j}\left(1-E_{\delta_{i}j}^{\left(t+1\right)}\right)},$$

(6) $$\beta^{\left(t+1\right)}=\frac{\sum_{i}\sum_{j}E_{\delta_{i}j}^{\left(t+1\right)}(1-X_{ij})}{\sum_{i}\sum_{j}E_{\delta_{i}j}^{\left(t+1\right)}}.$$

In summary, the parameters (E,α,β) are sampled or updated by following three steps: (i) initialize *α* and *β* at time step *t *=* *0; (ii) calculate the posterior of $E^{\left(t+1\right)}$ and generate $E^{\left(t+1\right)}$ by sampling from the posterior; (iii) update *α* and *β* to get $\alpha^{\left(t+1\right)}$ and $\beta^{\left(t+1\right)}$; (iv) repeat Steps (i) and (iii) until the model converges. More details about parameter estimation are provided in Supplementary Methods.

### 2.4 Datasets

There are several factors that control the generation of scDNA-seq mutation data, these factors include *α*, *β*, *N*, *M* and *K*. To fully assess the performance of bmVAE, we simulate various datasets by specifying different combinations of (β,N,M,K). The default values are set to *α* = 0.01, *β* = 0.3, *N *=* *1000, *M *=* *1000 and *K *=* *20. For each of the controlling factors, the candidate values selected for simulation are {0.3, 0.4, 0.5, 0.6} for *β* (dataset D1), {500, 1000, 2000, 5000} for *N* (dataset D2) and M (dataset D3) and {20, 30, 40, 50} for K (dataset D4). We also generate two smaller datasets to examine the minimum number of cells and mutations required by bmVAE to make accurate inferences: N∈ {100, 200, 300, 400, 500}, *M *=* *100 and *K *=* *5 for dataset D5; *N *=* *500, M∈ {20, 30, 40, 50, 60} and *K *=* *5 for dataset D6. We repeat the simulation five times for each setting of the parameters, thus get a total of 130 datasets for testing. In addition, to mimic doublet events, 10% of cells in each simulation are randomly selected to generate doublets following the approaches in Chen *et al.* (2020) and Jahn *et al.* (2016). More details about the simulation procedure are given in the Supplementary Methods.

We also obtain a high-grade serous ovarian cancer (HGSOC) dataset (McPherson *et al.*, 2016; Roth *et al.*, 2016) and an IDH-mutant gliomas dataset (Ciccolella *et al.*, 2021a; Venteicher *et al.*, 2017) to further assess our method.

### 2.5 Performance evaluation

We compare bmVAE to four state-of-the-art methods including RobustClone (Chen *et al.*, 2020), BnpC (Borgsmüller *et al.*, 2020), AMC (Yu and Du, 2022) and SCClone (Yu *et al.*, 2022), and two baseline dimensionality reduction methods including PCA (Joliffe and Morgan, 1992) and t-SNE (van der Maaten and Hinton, 2008). We select these methods for evaluation as they are significantly different from each other in the way of clustering mutation data. When making comparison to PCA and t-SNE, we replace the first module (dimensionality reduction) of bmVAE with the PCA and t-SNE, while remain other components unchanged. More details about how each method is used are provided in the Supplementary Methods.

We use two performance metrics to compare different methods. First, given the predicted and ground truth labels of cells, adjusted rand index (ARI) is calculated to evaluate clustering accuracy. Second, by comparing the inferred and ground truth genotype matrices, we use genotyping accuracy to indicate how well a method recovers the underlying mutational states of single cells. All methods except RobustClone are run five times on each sample to get the best performance metrics.

### 2.6 Implementation

All layers of the VAE network are fully-connected layers. Specifically, the encoder is composed of two hidden layers each with M/5 and M/10 neurons and a 3D latent layer, and the decoder has a mirrored structure to the encoder (a coarse search of the architecture hyper-parameters is given in Section 3.1). We use Leaky Rectified Linear unit (LeakyReLU) activation function in intermediate layers of the encoder and decoder, while employ sigmoid activation function in the final layer of the decoder. The network weights are updated using ‘RMSprop’ algorithm with learning rate of 0.0001 and batch size of 64. We set the number of epochs to 300, 300 and 50 on the simulated, HGSOC and IDH-mutant gliomas datasets, respectively. The weight factor *λ* in training the VAE is set to 0.0001 (details on search of the *λ* values can be found in Section 3.1). We run all the experiments on a computational server with 128 GB RAM, 64 CPU cores and 1 GeForce RTX 2080 Ti GPU. bmVAE is implemented in PyTorch, and the source code is freely available at https://github.com/zhyu-lab/bmvae.

## 3 Results

### 3.1 Hyper-parameter search

We conduct a coarse search of the architecture hyper-parameters, such as number of layers and hidden nodes of each layer. Specifically, values of 2, 3 and 4 are tested for the number of layers of the encoder network, and the numbers of hidden nodes in each layer are set to M/5, M/10, M/15 and M/20, respectively. We compare the clustering results associated with different architecture hyper-parameters (as shown in Supplementary Fig. S1) on the simulated dataset D1, and the parameter setting of two hidden layers each with M/5 and M/10 hidden nodes gives the best results, therefore, we chose this architecture to analyze all datasets. In addition, we test different latent dimensions and compare the clustering and genotyping results. As shown in Supplementary Figure S2, setting latent dimension to three achieves the best performance, and we set latent dimension to three on all experiments.

We also investigate the effect of hyper-parameter *λ* on clustering results. Specifically, values in {1e−5, 5e−5, 0.0001, 0.0005, 0.001} are tested for *λ*, and the comparison of clustering results on the simulated dataset D1 is shown in Supplementary Figure S3. The results indicate setting *λ* to 0.0001 produces the highest ARI and genotyping accuracy, therefore, we use *λ *= 0.0001 on all experiments.

### 3.2 Results on simulated datasets

#### 3.2.1 bmVAE shows higher robustness to the change of FNR

**Fig. 2.**
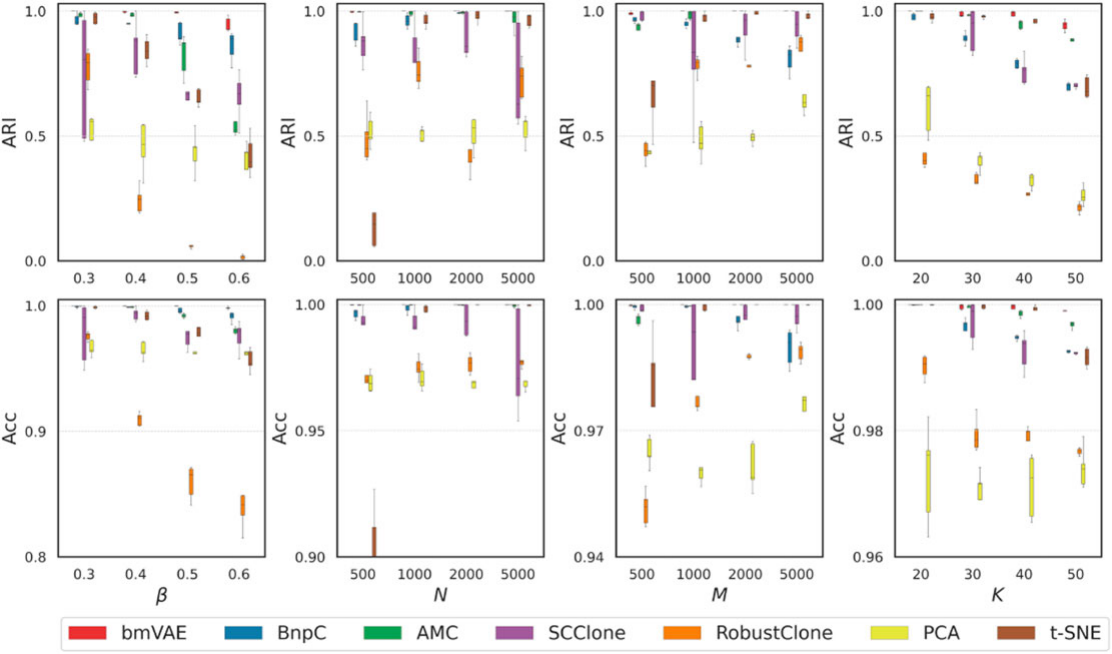
Performance comparison results on simulated datasets. The simulated FNR *β* changes from 0.3 to 0.6, number of cells *N* and number of mutations *M* change from 500 to 5000 and number of clusters *K* ranges from 20 to 50. Two performance metrics including ARI for clustering and genotyping accuracy are employed to assess the methods

We first assess bmVAE on the simulated dataset D1 where the FNR *β* is in {0.3, 0.4, 0.5, 0.6}. An example of the clustering results of bmVAE is depicted in Supplementary Figure S4. The performance comparison results (Fig. 2) show bmVAE achieves the highest accuracy on both clustering and genotyping compared to other methods. For instance, with *β* = 0.3, bmVAE performs the best with mean ARI of >0.999 and genotyping accuracy of >0.999, AMC outperforms other existing methods by achieving mean ARI of 0.98 as well as genotyping accuracy of >0.999, and BnpC also exhibits good performance with corresponding metric values of 0.966 and 0.999, whereas RobustClone and SCClone show lower accuracy. By comparison, t-SNE gets similar performance to BnpC, while dimensionality reduction using PCA does not provide similar results as bmVAE, indicating PCA is less effective in clustering binary data. When *β* increases to 0.6, the performance of each method degrades as the information related to clonal composition is severely attenuated by FN errors. Our method still gets higher accuracy than other methods on the highly disturbed binary data, it delivers average ARI of 0.947, while the ARIs of BnpC, AMC, SCClone, RobustClone, PCA and t-SNE are 0.86, 0.532, 0.658, 0.016, 0.389 and 0.421, respectively. An example of the clustering results with bmVAE, PCA and t-SNE when *β* = 0.6 is given in Supplementary Figure S5, and the results clearly show the superior performance of bmVAE in learning latent representations of the cells. The results also suggest RobustClone is sensitive to FN errors, thus its performance degrades significantly when *β* increases.

We also make a comparison between bmVAE and BnpC in estimating the FNR. The results in Supplementary Figure S6 show the predictions of bmVAE are closer to the ground truth value, while BnpC tends to underestimate the FNR, which may explain the better performance gained by bmVAE in both clustering and genotyping.

#### 3.2.2 bmVAE performs well on different-sized datasets

We evaluate the robustness of bmVAE to change in data size by applying it to the simulated datasets D2 and D3 where the number of cells *N* or mutations *M* is in {500, 1000, 2000, 5000}.

The results on dataset D2 (Fig. 2) suggest bmVAE’ performance is robust to the change of number of cells. When mutation data of more than 1000 cells are exploited, bmVAE is able to accurately decipher the clonal composition (an example of the clustering results is given in Supplementary Fig. S7) and recover the mutational profiles of cells, implying bmVAE effectively captures underlying evolutional patterns in latent space and maps them into distinct clusters. bmVAE also shows good runtime efficiency, and only requires ∼7 min per sample when handling large genotype matrix with *N *=* *5000. BnpC achieves similar accuracy to bmVAE when N≥ 2000, while it uses ∼100 min to process a sample with *N *=* *5000. Compared to bmVAE and BnpC, AMC suffers from slightly degraded performance as the number of cells increases, and SCClone’ accuracy fluctuates significantly on large datasets, which indicates it may frequently converge to suboptimal solutions. Same to bmVAE and BnpC, RobustClone yields improved genotyping accuracy when more cells are used for analysis. As RobustClone first recovers genotypes of the cells and then finds cell clusters, the improvement on genotyping accuracy does not promise an elevation on clustering accuracy. Interestingly, increasing the number of cells appreciably enhances the performance of t-SNE in clustering cells, thus enables improved genotyping results. For instance, the mean ARI of t-SNE increases from 0.208 at *N *=* *500 to 0.965 at *N *=* *5000. By comparison, PCA is less effective than t-SNE as the clustering accuracy does not change significantly across different *N* values.

We proceed to investigate how well bmVAE and other methods perform when the number of mutations changes. The results in Figure 2 show bmVAE, AMC, PCA, t-SNE and RobustClone get elevated accuracy when the number of mutations increases. For instance, mean clustering accuracy of t-SNE improves from 0.681 at *M *=* *500 to 0.983 at *M *=* *5000, and bmVAE consistently achieves >0.98 ARI across different *M* values. AMC performs similarly to bmVAE when *M *>* *1000. The mutational difference between tumor clones is enhanced with the increased number of mutations; therefore, the mutational patterns that distinguish different clones can be represented more effectively in latent space, which may contribute as a main factor resulting in the performance boost of bmVAE, PCA and t-SNE. As AMC and RobustClone employ PCA based methods to recover the genotypes of cells, their genotyping accuracies also improve with the number of mutations. By comparison, BnpC shows degraded accuracy when the number of mutations increases (its clustering accuracy decreases from 0.969 at *M *=* *500 to 0.807 at *M *=* *5000), and SCClone performs better than RobustClone and PCA. As BnpC jointly infers the cell clusters and their genotypes, it generally yields better clustering results than RobustClone especially on datasets with ≤1000 mutations.

To determine the minimum number of cells and mutations required by bmVAE to make accurate inferences, we further evaluate bmVAE on the simulated datasets D5 and D6. The number of cells *N* ranges from 100 to 500 for D5 and the number of mutations *M* varies from 20 to 60 for D6. The results in Supplementary Figure S8 imply with ≥200 cells bmVAE is able to accurately recover the clonal structure and estimate the genotypes. In addition, the results in Supplementary Figures S9 and S10 show with >30 mutations bmVAE achieves >0.91 median clustering accuracy and >0.995 median genotyping accuracy. Taken together, we recommend using bmVAE on datasets with at least 200 cells and 30 mutations.

#### 3.2.3 bmVAE is able to better detect clusters

We benchmark all methods on the simulated dataset D4, where the number of clusters *K* changes from 20 to 50, to check how data heterogeneity affects the clustering and genotyping accuracy. The number of cells and mutations are set to 2000. It is observed from Figure 2 that all methods tend to yield less accurate clustering results on datasets with higher data heterogeneity. For instance, the mean ARIs of bmVAE, BnpC, AMC, SCClone, RobustClone and t-SNE are 0.992, 0.968, 0.991, 0.991, 0.424 and 0.978 at *K *=* *20, while decrease to 0.943, 0.698, 0.893, 0.709, 0.213 and 0.694 at *K *=* *50, respectively. Compared to other methods, bmVAE appreciably gets higher clustering accuracy across different values of *K*, and also consistently maintains better genotyping results especially when *K *=* *50. The results also suggest PCA and t-SNE are less effective than bmVAE in learning latent representations of cells from data with high heterogeneity.

To further assess how well each method performs in predicting clonal composition, we make a comparison between the estimated and ground truth number of clusters, and the results are shown in Supplementary Figure S11. On simple datasets with 20 clusters, bmVAE, BnpC, AMC, SCClone and t-SNE are able to report the number of clusters close to the ground truth, while RobustClone and PCA consistently underestimate the number of clusters in all cases, which explains their lower clustering and genotyping accuracy. On highly heterogeneous datasets with more than 30 clusters, all methods except AMC tend to underestimate the number of clusters whereas AMC overestimates it, and bmVAE still yields better predictions that are closer to the true value.

### 3.3 Results on real datasets

#### 3.3.1 HGSOC dataset

The HGSOC dataset describes binary genotypes of 420 cells at 43 genomic loci. The cells are taken from omentum (Om), left ovary (LOv) and right ovary (ROv). Previous studies have applied RobustClone, BnpC and SCClone to this dataset for detecting tumor clones, and all of the three methods identify five clusters.

**Fig. 3.**
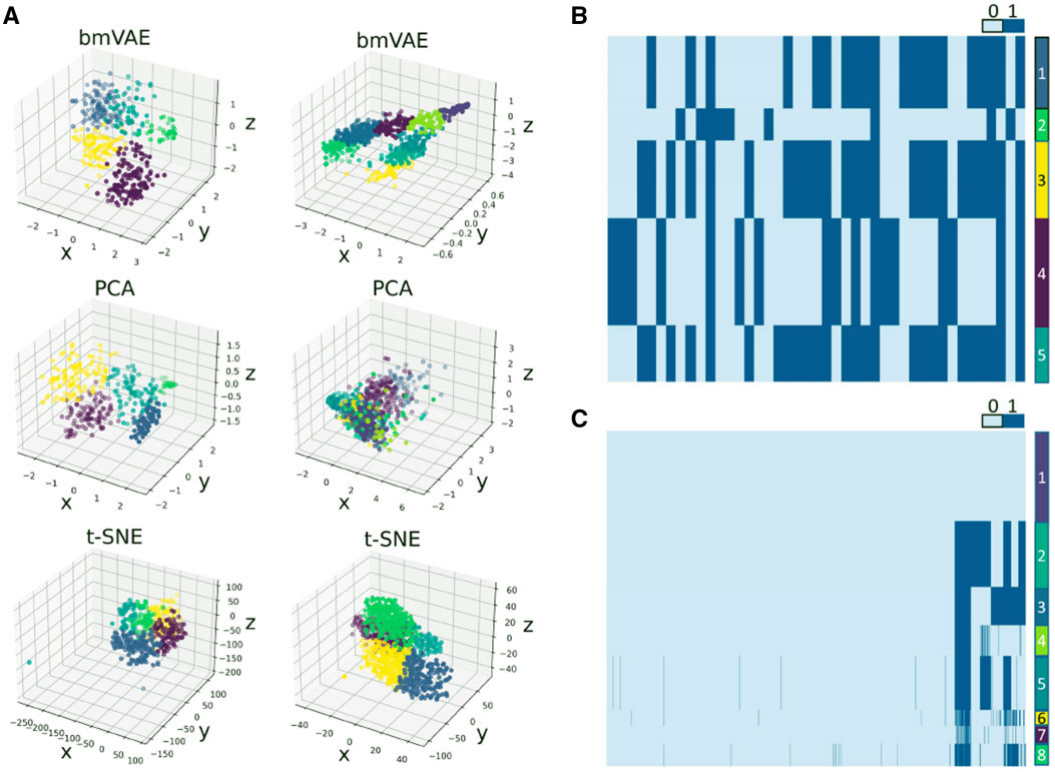
Clustering and genotyping results on real datasets. (**A**) Clustering results of bmVAE, PCA and t-SNE in low-dimensional space. On the HGSOC dataset (left subplots), bmVAE, PCA and t-SNE find five clusters. On the IDH-mutant gliomas dataset (right subplots), bmVAE clusters the cells into eight subpopulations, PCA finds seven clusters and t-SNE captures five clusters; (**B**) genotypes of subpopulations estimated by bmVAE on HGSOC dataset; (**C**) genotypes of subpopulations estimated by bmVAE on the IDH-mutant gliomas dataset

We check if bmVAE is able to give similar findings as RobustClone, BnpC and SCClone on this dataset. Interestingly, bmVAE clusters the cells into five subpopulations by learning latent representations of the cells in a 3D space (Fig. 3A and B), and estimates the error rates as FPR = 0.028 and FNR = 0.338. Similar to the clustering results observed on the simulated dataset D6 (Supplementary Fig. S10), the distances between distinct clusters are less significant in the latent space, while they are still clearly separated and keep a reasonable margin with each other. The clustering results show there are apparent mutational differences between cells originating from different tumor sites. For instance, Cluster 3 mainly consists of ROv and Om cells taken from metastatic tumor sites, Cluster 4 mainly contains LOv cells that originate from the primary tumor site, while Cluster 5 only encompasses ROv and Om cells (Supplementary Fig. S12), and our findings are similar to the reported results of BnpC. In addition, we further examine if PCA and t-SNE are also effective in learning low-dimensional representations of cells on this dataset. The results show both PCA and t-SNE detect five clusters (Fig. 3A). Compared to PCA and t-SNE, bmVAE produces clearer boundary between distinct clusters in the latent space.

#### 3.3.2 IDH-mutant gliomas dataset

Finally, we apply bmVAE to the IDH-mutant glioma dataset that contains a 926 × 1392 genotype matrix. bmVAE takes 2 min to finish the clustering and genotyping pipeline, identifies a normal population and seven tumor clones (Fig. 3A and C). The estimated error rates are FPR = 0.015 and FNR = 0.727, which suggests the data may be heavily confounded by ADO.

We also obtain results of BnpC and RobustClone on this dataset, to evaluate the similarity of the tumor clonal structures captured by different methods. BnpC finds as high as 33 clusters, estimates the FPR as 0.011 and FNR as 0.77. Compared to bmVAE, BnpC yields similar predictions of the error rates, but subdivides the cells into more small clusters. Conversely, RobustClone fails to identify any tumor clones due to high sparsity of the data and assigns all cells to normal population, which is in concordance with the results observed on simulated dataset D1 that RobustClone suffers from severe performance degradation on high FNR data. AMC reports 18 clusters on this dataset while estimates the error rates as FPR = 0.0096 and FNR = 0.81 (Yu and Du, 2022). These results, to some extent, are similar to those of bmVAE and BnpC. Moreover, we use PCA and t-SNE to reduce the dimensionality, and cluster cells based on the projected low-dimensional data. PCA identifies seven clusters on this dataset, while the inferred clusters are not clearly separated in the latent space (as shown in Fig. 3A). t-SNE finds five clusters, and may underestimate the underlying number of clusters as the results on simulated dataset D1 show t-SNE is less effective in clustering cells from high FNR data.

## 4 Discussion

Clustering single-cell mutation data comes as an important paradigm of analyzing scDNA-seq data and can be treated as a preprocessing task to reduce data size before inference of tumor phylogeny. In this article, we develop a VAE-based method for this purpose, and our method is specifically designed to process binary data. One advantage of bmVAE is the effective learning of latent representation of cell via the VAE model. VAE has been successfully applied to scRNA-seq data for dimensionality reduction and shows better performance compared to conventional methods, such as PCA and t-SNE. This encourages us to introduce a new VAE model for single-cell binary mutation data, and infer the low-dimensional representation of mutational patterns that characterize distinct tumor clones, which facilitates clustering of cells with high efficiency. Another advantage of bmVAE is the efficient estimation of clonal genotypes via Gibbs sampling method. Given the learned labels of cells, the clonal genotypes as well as error rates are efficiently inferred under a Gibbs sampling scheme. Unlike the pipeline adopted by RobustClone that first recovers the genotypes of cells and then finds clusters of the cells, our method employs a contrary flow and gains higher robustness in both clustering and genotyping.

Despite of the novelty mentioned above, there are still two limitations in using bmVAE. First, some of the clusters may result from doublet cells, while bmVAE does not distinguish between them and true clonal clusters, which may affect the inference accuracy when doublets frequently occur in the data. Incorporating the effects of doublets into the downstream analysis may deliver more accurate predictions of the clonal genotypes, while this requires more complex statistical models. Second, the lineage relationship between distinct clones is defined in the underlying tumor phylogeny that imposes a constraint on the mutational states of clones, while bmVAE does not consider tumor phylogeny when estimating the genotypes, which may lead to biased predictions. Joint estimation of clonal genotypes and tumor phylogeny is an effective way to further improve the inference accuracy of bmVAE. As mutations that appear in the same edge or node of the phylogeny tree can be grouped together (Yu and Du, 2022), biclustering can be used to jointly find blocks of cells and blocks of mutations, which helps to yield more accurate predictions of tumor clones. We plan to investigate this research direction in near future.

In summary, we introduce bmVAE for clustering single-cell mutation data based on dimensionality reduction. bmVAE can be used as a plug-in module in the pipeline of tumor phylogeny inference to reduce the data size, thus improves the efficiency and accuracy of tumor tree reconstruction.

## Supplementary Material

btac790_Supplementary_Data

## Data Availability

The data underlying this article will be shared on reasonable request to the corresponding author.
