# Supplementary material for: bmVAE: a variational autoencoder method for clustering single-cell mutation data
Source: Bioinformatics. 2022 Dec 7;39(1):btac790. doi: 10.1093/bioinformatics/btac790 (PMC9825778; doi:10.1093/bioinformatics/btac790)
Supplement: btac790_Supplementary_Data [file btac790_supplementary_data.pdf]

bmVAE: a variational autoencoder method for clustering single-cell mutation data

Supplementary Material

Jiaqian Yan[1], Ming Ma[1] and Zhenhua Yu[1,2*]

[1]School of Information Engineering and [2]Collaborative Innovation Center for Ningxia Big Data and Artificial Intelligence Co-founded by Ningxia Municipality and Ministry of Education, Ningxia University, Yinchuan 750021, China

*To whom correspondence should be addressed.

Contact: zhyu@nxu.edu.cn

Contents

1. Supplementary Methods **2**

1.1. Estimating the error rates 2

1.2. Simulating scDNA-seq mutation data 2

1.3. Parameter settings 3

2. Supplementary Figures **4**

1. Supplementary Methods

1.1. Estimating the error rates

Suppose X is the input $N \times M$ binary genotype matrix (N represents the number of cells and M is the number of mutations), $\Delta=(\delta_1, \delta_2, \ldots, \delta_N)$ is a vector to indicate the inferred labels of cells, E is a $K \times M$ binary matrix to represent the mutational profiles of K clusters, false positive rate (FPR) and false negative rate (FNR) are α and β, respectively, then the likelihood at the t-th iteration of Gibbs sampling is given by:

$$p(X \mid E^{(t)}, \alpha, \beta) = \prod_{i=1}^{N} \prod_{j=1}^{M} \left((1-\beta)^{X_{ij}} \beta^{1-X_{ij}} \right)^{E_{\delta_i j}^{(t)}} \left((1-\alpha)^{1-X_{ij}} \alpha^{X_{ij}} \right)^{1-E_{\delta_i j}^{(t)}} \tag{1}$$

We want to infer the maximum likelihood estimations of α and β:

$$\begin{aligned} (\alpha^{(t)}, \beta^{(t)}) &= \underset{\alpha,\beta}{\arg\max} \log\left(p(X \mid E^{(t)}, \alpha, \beta) \right) \\ &= \underset{\alpha,\beta}{\arg\max} \sum_{i=1}^{N} \sum_{j=1}^{M} \left(\begin{array}{l} E_{\delta_i j}^{(t)} \left(X_{ij} \log(1-\beta) + (1-X_{ij}) \log(\beta) \right) \\ +(1-E_{\delta_i j}^{(t)}) \left((1-X_{ij}) \log(1-\alpha) + X_{ij} \log(\alpha) \right) \end{array} \right) \end{aligned} \tag{2}$$

Solving the above optimization function yields following solutions:

$$\alpha^{(t)} = \frac{\sum_i \sum_j (1-E_{\delta_i j}^{(t)}) X_{ij}}{\sum_i \sum_j (1-E_{\delta_i j}^{(t)})} \tag{3}$$

$$\beta^{(t)} = \frac{\sum_i \sum_j E_{\delta_i j}^{(t)} (1-X_{ij})}{\sum_i \sum_j E_{\delta_i j}^{(t)}} \tag{4}$$

The Gibbs sampling procedure continues until the mean absolute difference between the posteriors of E at two consecutive iterations is less than a predefined threshold (10^{-6}).

1.2. Simulating scDNA-seq mutation data

We employ a similar procedure as proposed in (Ross and Markowetz, 2016) to generate scDNA-seq mutation data, that is first emulating a phylogenetic tree and then sampling cells from the tree. We initialize the tree by randomly choosing a clone as the child of the root, and iteratively add an edge $<p, c>$ to the tree, where p represents a uniformly sampled non-root node of the tree and c denotes a randomly selected clone that has not yet added to the tree. For assigning the mutations to the edges, we first attach a mutation to each of the edges, then randomly assign remaining mutations to the edges. This yields a clonal tree where each node except the root represents a tumor clone and each edge is labeled with the mutations that are further acquired by the child clone. For assigning cells to nodes of the tree, we first attach a cell to each of the nodes, then iteratively attach remaining cells to the nodes of the tree, and the probability that a node is selected as

the attachment point is proportional to the current size of the node, which enables different sized clones to be generated. Finally, the ground truth genotypes of cells are deduced from the phylogenetic tree, and further processed to generate observed data according to simulated FPR and FNR.

When mimicking doublet cells, logical OR (||) operator is applied to each mutation to generate a doublet from two cells.

1.3. Parameter settings

The specific parameter settings of each method are as follows: 1) RobustClone and SCClone are tested under the default parameters; 2) for BnpC, we set the runtime to $N/50$ minutes according to the reported results in BnpC paper (Borgsmüller, et al., 2020); 3) we first use AMC to cluster mutations, and then employ SCITE (Jahn, et al., 2016) to reconstruct the mutation tree where each node represents a cell cluster. The mutational states of each tumor clone are deduced from the inferred tree; and 4) for t-SNE, we first employ PCA to reduce the dimension to 50, 10 and 50 on simulated, HGSOC and IDH datasets, respectively, then use t-SNE to reduce the dimension to the desired latent dimension. For each dataset, the perplexity parameters in {10, 20, 30, 40, 50} are tested and the solution that produces the highest ARI score is selected as the optimal solution on simulated datasets, while on real datasets we manually select the solution that yields the best separation of the cells.

Reference

Borgsmüller, N., *et al.* (2020) BnpC: Bayesian non-parametric clustering of single-cell mutation profiles, *Bioinformatics*, **36**, 4854-4859.

Jahn, K., Kuipers, J. and Beerenwinkel, N. (2016) Tree inference for single-cell data, *Genome Biology*, **17**, 86.

Ross, E.M. and Markowetz, F. (2016) OncoNEM: inferring tumor evolution from single-cell sequencing data, *Genome Biology*, **17**, 69.

2. Supplementary Figures

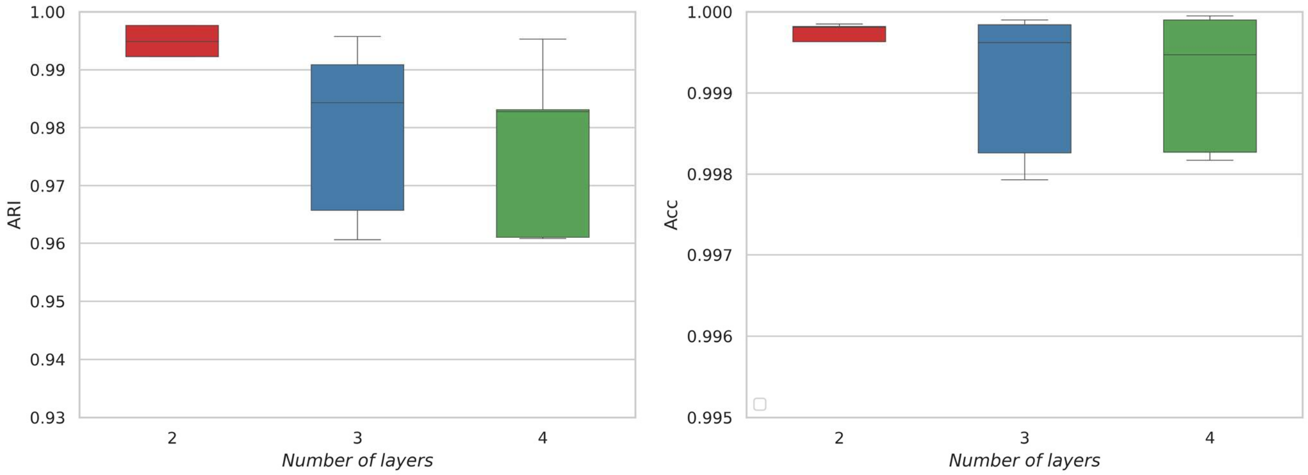

Fig. S1. Comparison between different architectures of the variational autoencoder (VAE) adopted in bmVAE. Values of 2, 3 and 4 are tested for the number of layers of the encoder network (decoder network has a mirrored structure to the encoder), and the numbers of hidden nodes in each layer are set to *M*/5, *M*/10, *M*/15 and *M*/20, respectively (here *M* denotes the number of mutations). The comparison is conducted on the simulated dataset D1 where genotype matrices with $\beta = 0.3$ are used for evaluation.

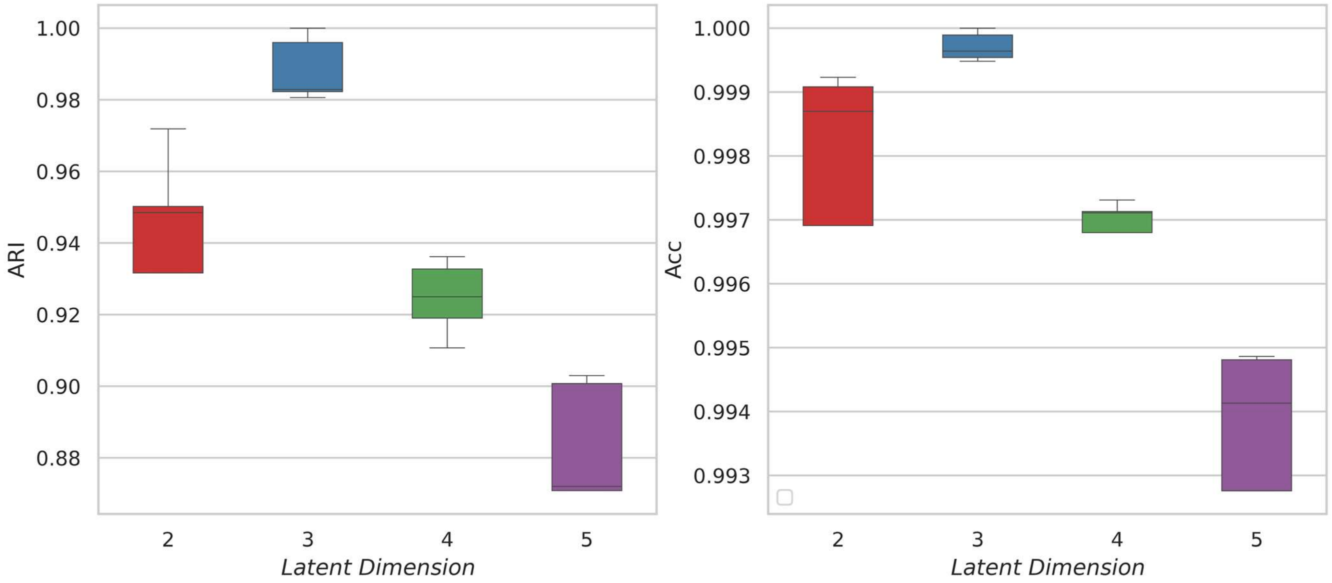

Fig. S2. Comparison between different latent dimensions of the VAE. Values of 3, 4 and 5 are tested for the latent dimensions. The comparison is conducted on the simulated dataset D1 where genotype matrices with $\beta = 0.3$ are used for evaluation.

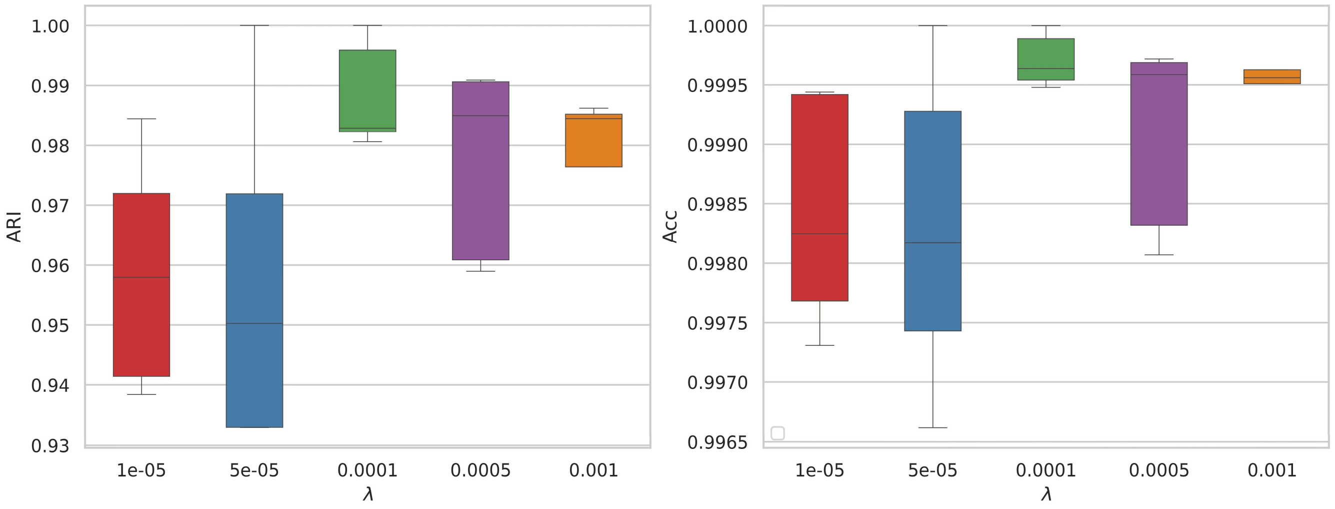

Fig. S3. Comparison between different values of the hyper-parameter λ. Values in {1e-5, 5e-5, 0.0001, 0.0005, 0.001} are tested for λ. The comparison is conducted on the simulated dataset D1 where genotype matrices with $\beta = 0.3$ are used for evaluation.

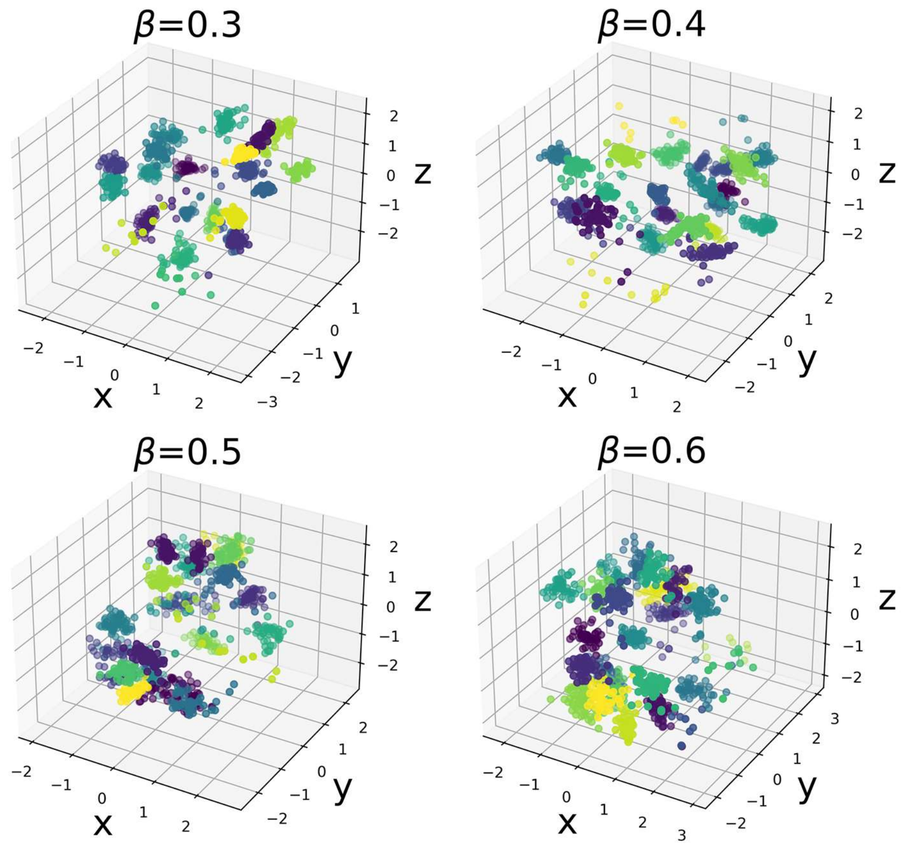

Fig. S4. An example of the clustering results with bmVAE on simulated dataset D1. When β increases from 0.3 to 0.6, bmVAE performs well in identifying distinct clusters.

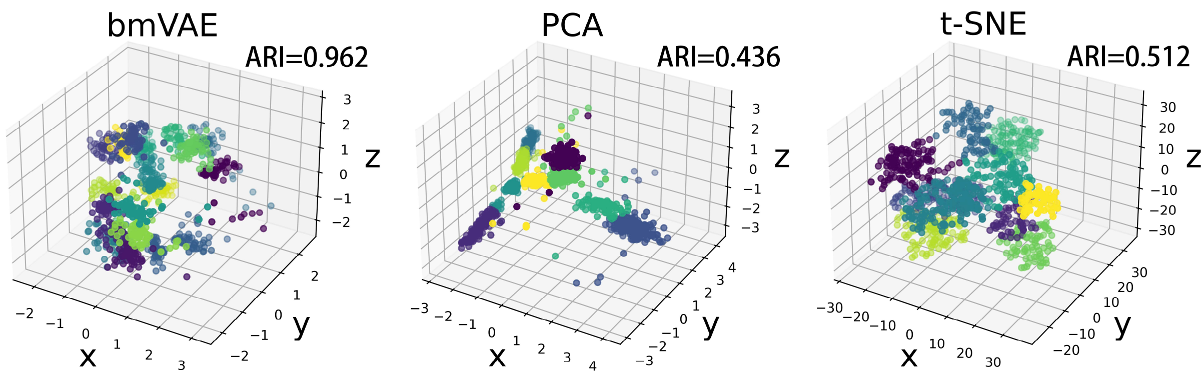

Fig. S5. An example of the clustering results with bmVAE, PCA and t-SNE when $\beta = 0.6$. Compared to PCA and t-SNE, bmVAE is more effective in learning the latent features of cells, thus delivers more accurate clustering results.

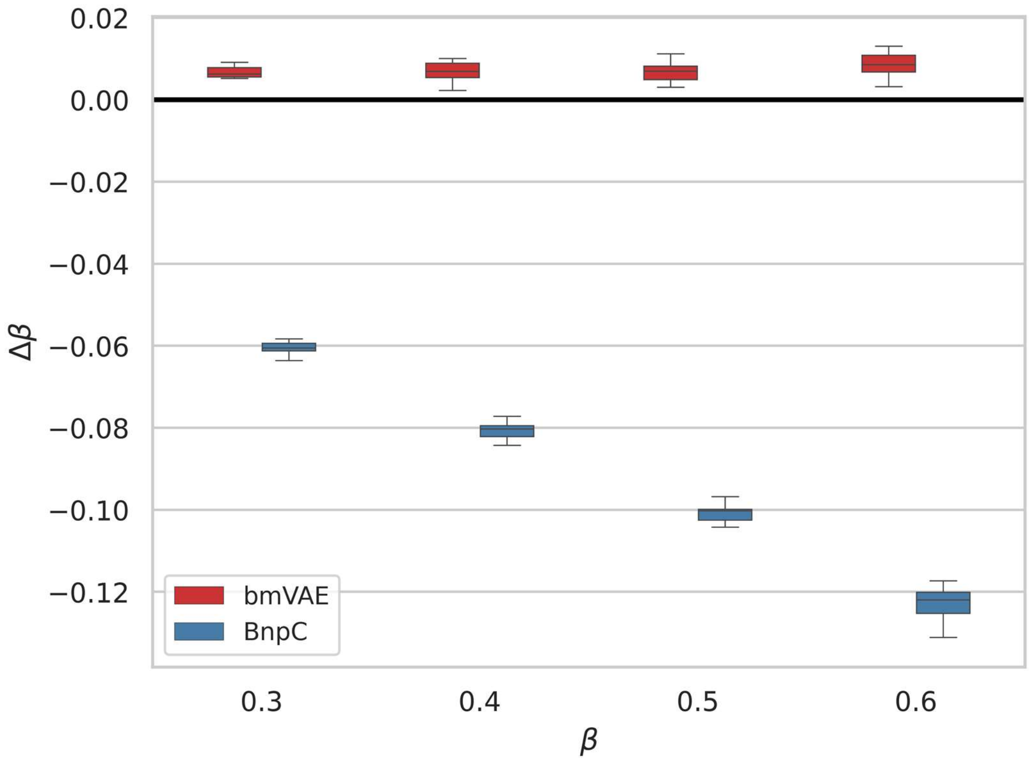

Fig. S6. FNR estimation results of bmVAE and BnpC on simulated dataset D1. $\Delta\beta$ denotes the difference between the predicted and ground truth FNRs.

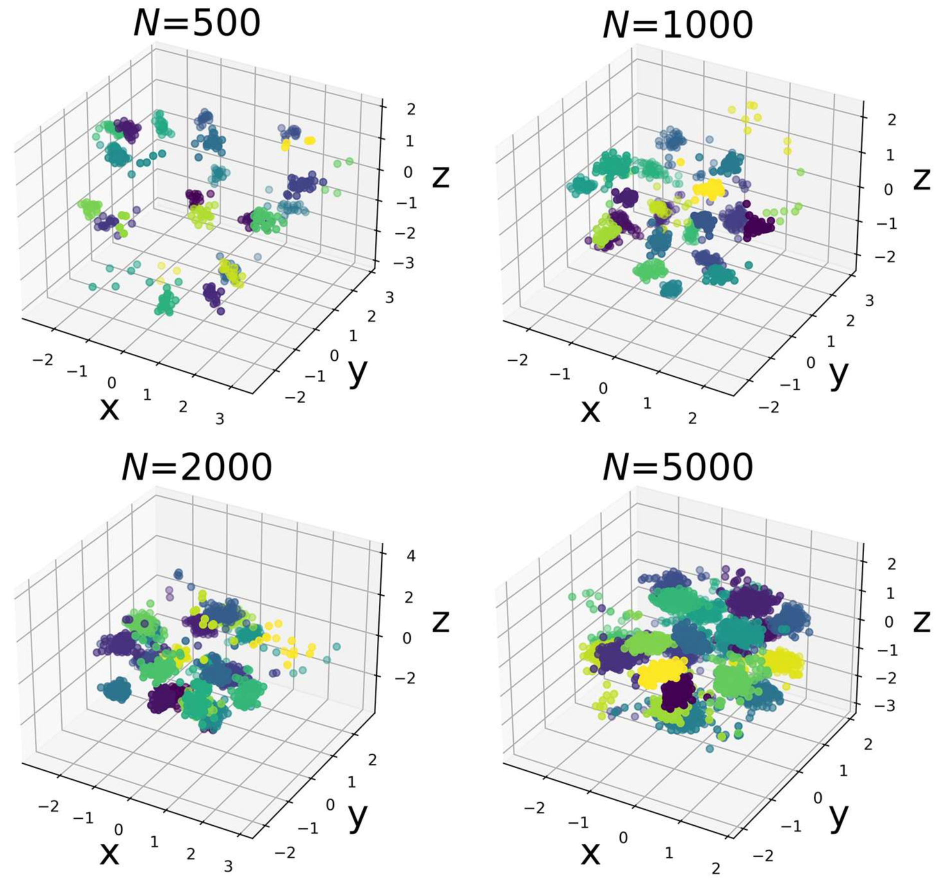

Fig. S7. An example of the clustering results with bmVAE on simulated dataset D2. The number of cells N increases from 500 to 5000.

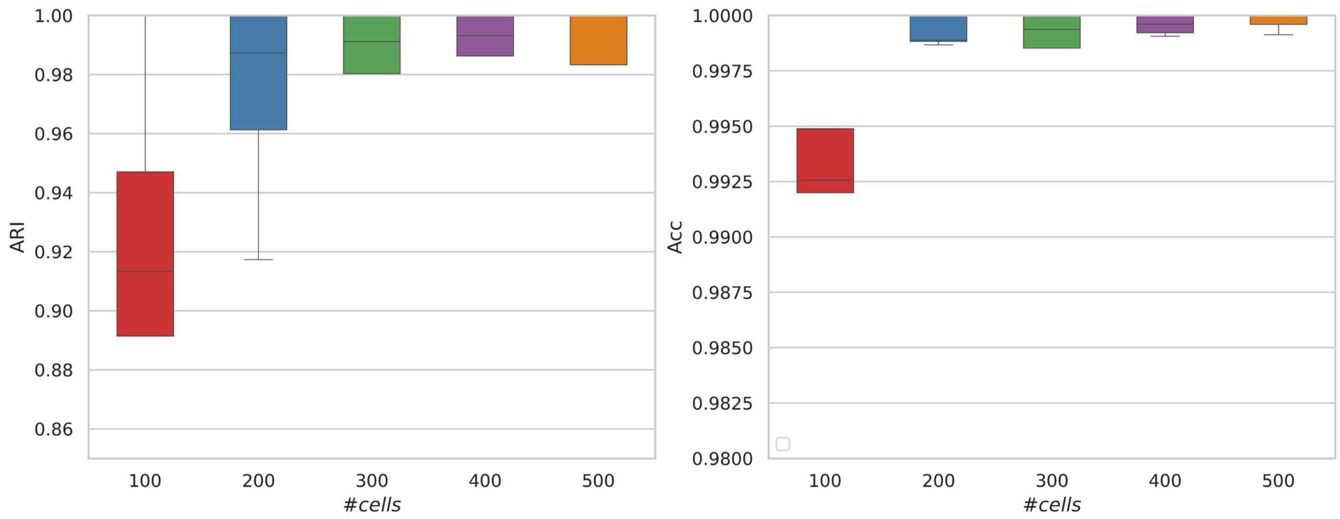

Fig. S8. Clustering and genotyping performance of bmVAE on the simulated dataset D5. The number of cells ranges from 100 to 500.

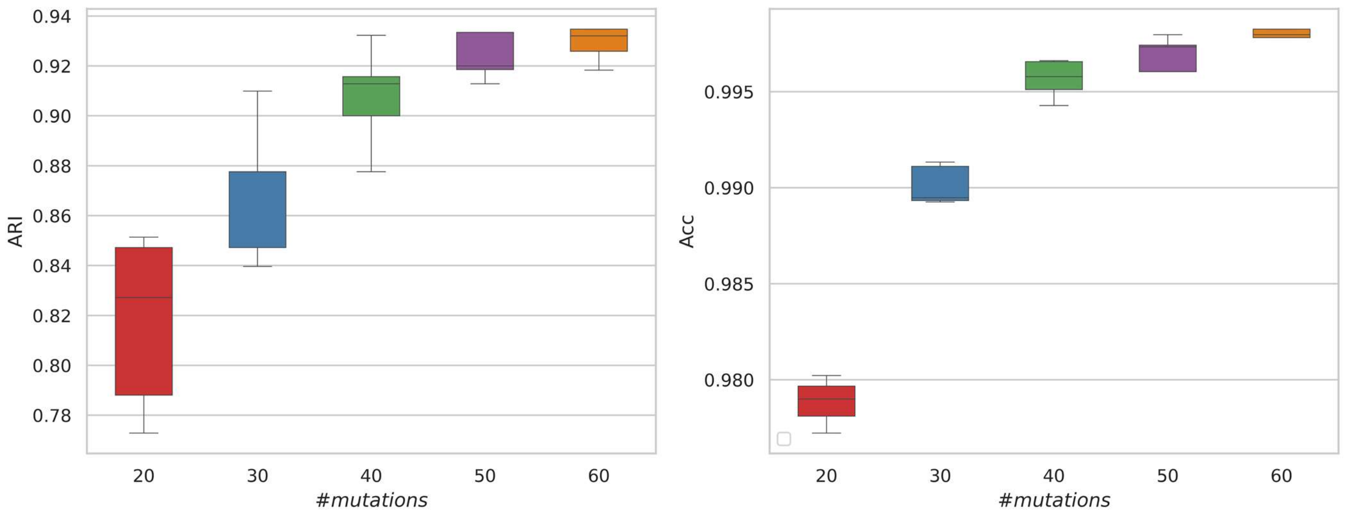

Fig. S9. Clustering and genotyping performance of bmVAE on the simulated dataset D6. The number of mutations ranges from 20 to 60.

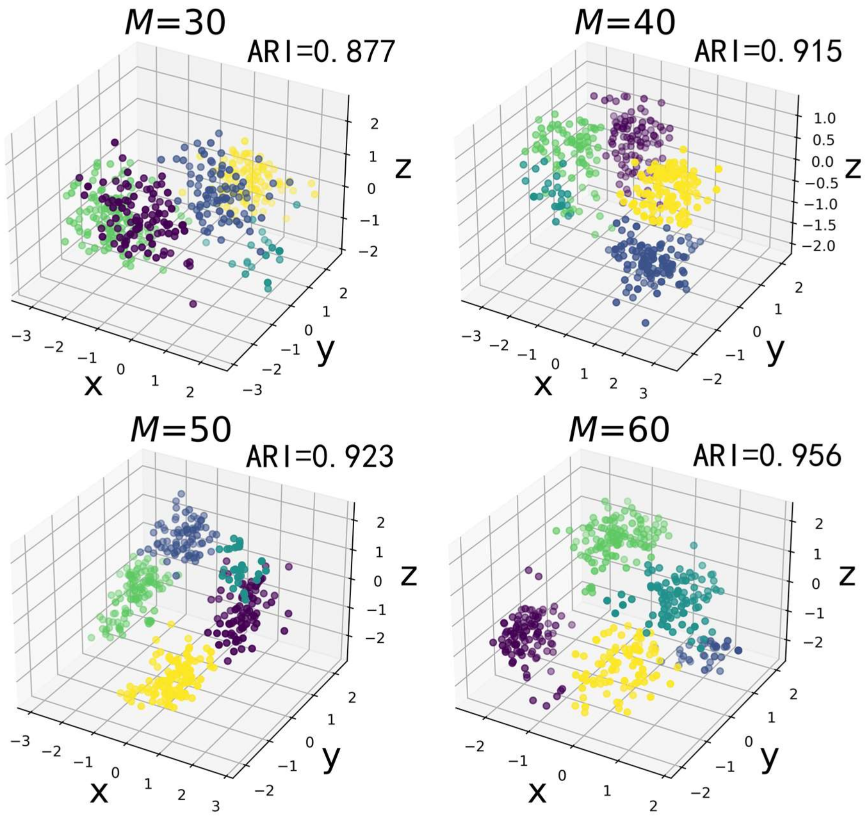

Fig. S10. Clustering results of bmVAE on the simulated dataset D6. When the number of mutations increases, bmVAE yields good separation of the cells in latent space.

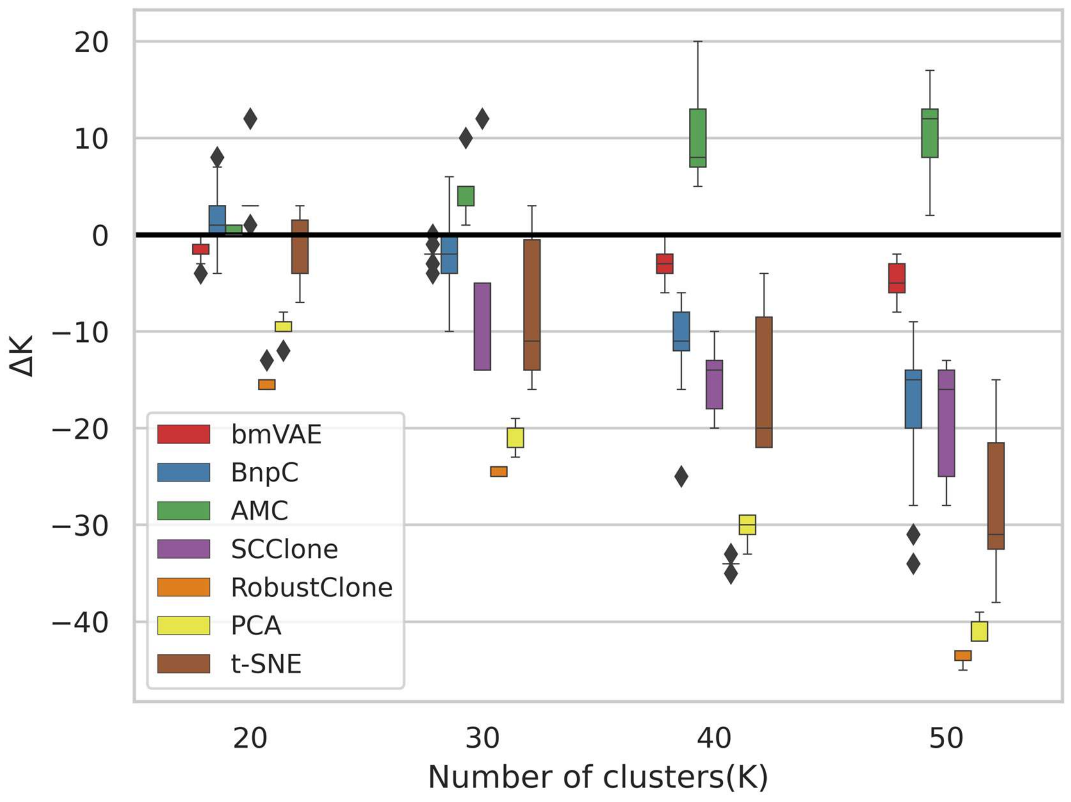

Fig. S11. The number of clusters predicted by the methods on simulated dataset D4. ΔK denotes the difference between the predicted and ground truth number of clusters.

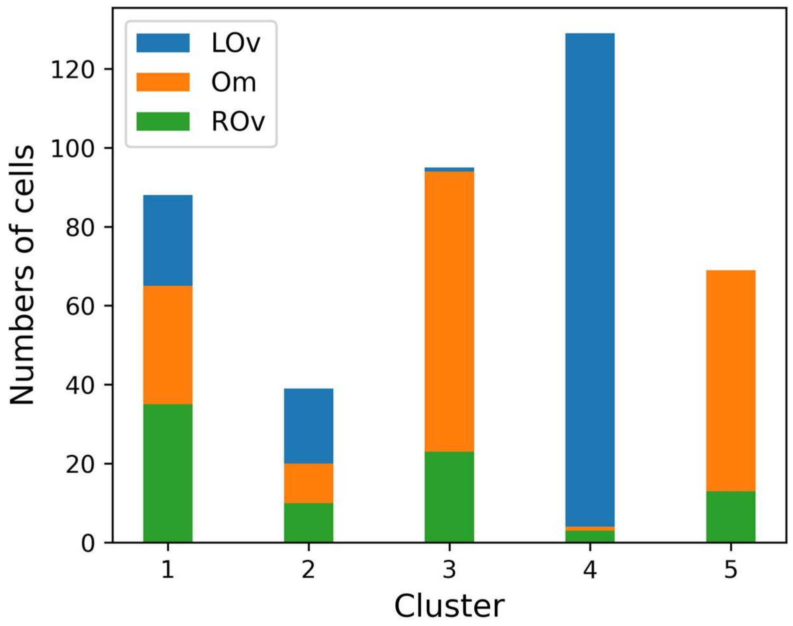

Fig. S12. Distribution of the cells across distinct clusters on HGSOC dataset. The HGSOC dataset contains a 420×43 genotype matrix, bmVAE finds five clusters on this dataset. Cluster 3 mainly consists of ROv and Om cells taken from metastatic tumor sites, cluster 4 mainly contains LOv cells that originate from the primary tumor site, while cluster 5 only encompasses ROv and Om cells.
